# A Novel *STAT3* Gain-of-Function Mutation in Fatal Infancy-Onset Interstitial Lung Disease

**DOI:** 10.3389/fimmu.2022.866638

**Published:** 2022-05-23

**Authors:** Mengyue Deng, Yue Li, Yulu Li, Xiaolan Mao, Han Ke, Weiling Liang, Xiaoguang Lei, Yu-Lung Lau, Huawei Mao

**Affiliations:** ^1^ Department of Pediatric Research Institute, Children’s Hospital of Chongqing Medical Univeristy, National Clinical Research Center for Child Health and Disorders, Ministry of Education Key Laboratory of Child Development and Disorders, Chongqing Key Laboratory of Child Infection and Immunity, Chongqing, China; ^2^ Beijing National Laboratory for Molecular Sciences, Key Laboratory of Bioorganic Chemistry and Molecular Engineering of Ministry of Education, Department of Chemical Biology, College of Chemistry and Molecular Engineering, Synthetic and Functional Biomolecules Center, and Peking-Tsinghua Center for Life Sciences, Peking University, Beijing, China; ^3^ Department of Pediatrics, The University of Hong Kong-Shenzhen Hospital, Shenzhen, China; ^4^ Department of Paediatrics and Adolescent Medicine, The University of Hong Kong, Hong Kong, Hong Kong SAR, China

**Keywords:** *STAT3*, gain-of-function mutation, interstitial lung disease, autoimmune disease, STAT3 inhibitor, JAK inhibitor

## Abstract

Signal transducer and activator of transcription 3 (*STAT3*) gain-of-function (GOF) mutations cause early-onset immune dysregulation syndrome, characterized by multi-organ autoimmunity and lymphoproliferation. Of them, interstitial lung disease (ILD) usually develops after the involvement of other organs, and the onset time is childhood and beyond rather than infancy. Here, we reported a patient who presented with fatal infancy-onset ILD, finally succumbing to death. Next-generation sequencing identified a novel heterozygous mutation in *STAT3* (c.989C>G, p.P330R). Functional experiments revealed it was a gain-of-function mutation. Upon interleukin 6 stimulation, this mutation caused a much higher activation of STAT3 than the wild-type control. In addition, the mutation also activated STAT3 under the steady state. The T helper 17 cell level in the patient was significantly higher than that in normal controls, which may contribute to the autoimmune pathology caused by the STAT3^P330R^ mutation. Apart from Janus kinase (JAK) inhibitors, we also provided experimental evidence of a STAT3 selective inhibitor (Stattic) effectively suppressing the activation of mutant STAT3 *in vitro*. Collectively, our study expanded the clinical spectrum of *STAT3* GOF syndrome. *STAT3* GOF mutation appears as a new etiology of ILD and should be considered in patients with early-onset ILDs. In addition to JAK inhibitors, the specific STAT3 inhibitor would be an appealing option for the targeted treatment.

## Introduction

Signal transducer and activator of transcription 3 (STAT3) belongs to the STATs family of transcription factors (1). In the cytokine signaling cascade, STAT3 is tyrosine phosphorylated by Jak kinase (JAK) and translocates as a dimer into the nucleus, where it modulates the expression of target genes (1). *STAT3* is involved in multiple physiological and developmental processes, including immune cell activation and differentiation (2). Distinct germline mutations in *STAT3* cause either loss-of-function or gain-of-function (GOF) and present with different immune-related clinical phenotypes (3). *STAT3* GOF mutations cause early-onset immune dysregulation syndrome, characterized by multi-organ autoimmunity and lymphoproliferation (4, 5). The involved organs appear to occur sequentially, with the early onset of endocrine and gastrointestinal disorders preceding other organs (6). The onset age of interstitial lung disease (ILD) was childhood and substantially later than other clinical phenotypes (6).

The increased transcriptional activity is now proposed as the basis for the pathogenesis of *STAT3* GOF mutation (4, 5). However, not all *STAT3* GOF mutations affect STAT3 phosphorylation (4, 5, 7). Furthermore, in patients with *STAT3* GOF mutation, there is generally no consistent pattern of abnormal immune features. The increased, decreased, or normal levels of T helper 17 (Th17) cells and the presence or absence of regulatory T (Treg) cell abnormality have been reported (4, 5, 8, 9). The marked differences in the immune-phenotypes increase the difficulty of thoroughly studying the pathogenesis of autoimmunity.

There are no established treatment guidelines currently available for *STAT3* GOF disorders (10, 11). Both the immunosuppressive agents and targeted treatments have been reported. Whereas the former drugs were found to be overall ineffective, and instead, they increased the risk of infections (12–15). Targeted treatments such as JAK inhibitors and monoclonal antibodies against the interleukin 6 (IL-6) receptor have been proven to target the JAK-STAT pathway indirectly and shown promising results in *STAT3* GOF diseases (4, 16). However, the pan-JAK inhibitors, ruxolitinib and tofacitinib targeting JAK1/2 and JAK1/3, respectively, lack pathway selectivity. Direct STAT3 inhibitors may be a more appealing treatment option.

In this study, we identified a novel *de novo* heterozygous *STAT3* mutation in a patient with fatal infancy-onset ILD. Functional verification of the mutation was performed. We further examined the effects of both JAK inhibitors and specific STAT3 inhibitor on the activation of mutant STAT3. The relevant literature was also reviewed and analyzed.

## Materials and Methods

### The Patient and Healthy Subjects

The patient, available family members, and age-matched controls were included in this study. The project was approved by our Institutional Review Board and conducted according to the Declaration of Helsinki Principles. Informed consent was obtained from the parents to participate in this study.

### Targeted Next-Generation Sequencing

Due to the financial issue, whole-exome sequencing was not performed. Instead, targeted next-generation sequencing, including library construction, capture, and sequencing, was carried out at MyGenostics Gene Technologies (Beijing, China) using genomic DNA isolated from the whole blood of the patient and his parents. The panel was designed to cover the genes known to cause immune diseases.

### Protein Structure Modeling

All structure models were visualized using the molecular graphics program PyMol. The STAT3 structure (PDB ID: 6TLC) was obtained from the Research Collaboratory for Structural Bioinformatics Protein Data Bank (http://www.rcsb.org/). All heteroatoms were removed, and chain A was kept as a STAT3 wild type (WT) monomer. The STAT3 P330R structure was mutated using the PyMol mutagenesis tool. Hydrophobic surfaces were colored based on a normalized hydrophobicity scale using the Color_h script (https://pymolwiki.org/index.php/Color_h). The electrostatic potential was calculated using the PyMol vacuum electrostatics program.

### Molecular Dynamics Simulations

Molecular-dynamics (MD) simulations were carried out using Gromacs 5.0.7 on the STAT3^WT^ and STAT3^P330R^ monomer structures. The Amber ff14SB force field was used to model the protein, and the TIP3P water model was used to describe the solvent. Then, the system was energy minimized and equilibrated using first NPT and then NVT, followed by a 50 ns equilibrium simulation.

### Flow Cytometric Analysis of Phospho-STAT3

The patient was hospitalized in the inpatient ward and treated with steroid, hydroxychloroquine and antimicrobial agents. Peripheral blood was collected and peripheral blood mononuclear cells (PBMCs) were isolated by density centrifugation. The cells were resuspended in RPMI 1640 supplemented with penicillin, streptomycin, and L-glutamine along with 10% fetal calf serum. PBMCs were then stimulated for 15 min with 20 ng/ml IL-6 (Peprotech) or not. Following stimulation, cells were immediately fixed with PhosFlow Fix Buffer I (BD Biosciences) for 10 min at 37°C, then permeabilized using BD Phosflow Perm Buffer III (BD Biosciences) following the manufacturer’s instruction. Cells were then stained with anti-STAT3 (pY705) or isotype-matched control antibodies (BD Biosciences) for 1 hour. Samples were collected with FACSCanto II (BD Biosciences) and analyzed with FlowJo Software (TreeStar).

### Expression Plasmids

The human *STAT3* coding sequence was cloned from cDNA isolated from the whole blood using reverse transcription PCR. The primer pair was forward: 5’-cttggtaccgagctcggatccgccaccATGGCCCAATGGAATCAGCTA-3’; reverse: 5’-gaagggccctctagactcgagCATGGGGGAGGTAGCGCA-3’. The PCR product was then ligated into the pcDNA3.1-3×Flag-C vector between the BamHI and XhoI restriction sites using ClonExpress II One Step Cloning Kit (Vazyme). The mutation was introduced using Mut Express II Fast Mutagenesis Kit V2 (Vazyme) following the manufacturer’s instructions. The mutagenesis primer pair was forward 5’-CCTGCATGCgCATGCATCCTGACCGGCCCCTCGTC-3’; reverse 5’-AGGATGCATGcGCATGCAGGGCTGCCGCTCCACCAC-3’.

### Western Blot

Human embryonic kidney 293 (HEK293) cells (ATCC) were transfected with the WT or mutant *STAT3* plasmid or empty vector. The transfected cells were stimulated with IL-6 (20 ng/mL) for 15, 30, or 60 min. Total STAT3 protein (Cell Signaling Technology), phospho-STAT3 (Y705) (Cell Signaling Technology) and GAPDH (Proteintech) were measured by western blot. For dephosphorylation experiments, the transfected HEK293 cells were stimulated with IL-6 (20 ng/mL) for 30min and then harvested at different times (15, 30, 60, or 120 min) after IL-6 withdrawal.

### STAT3 Reporter Assay

HEK293 cells were transiently co-transfected with indicated *STAT3* expression plasmid and a STAT3-responsive firefly luciferase reporter plasmid (Beyotime) and a renilla luciferase reporter plasmid (Beyotime) using Lipofectamine 3000 reagent (Invitrogen) according to the manufacturer’s protocol. Twenty-four hours after the transfection, the cells were not or treated with 20 ng/mL IL-6 for 16 hours. The luciferase activity was measured using the Dual-Glo Luciferase Assay System (Promega).

### Intracellular Cytokine Staining

PBMCs were stimulated with 50 ng/ml phorbol myristate acetate (PMA) plus 500 ng/ml ionomycin for five hours in the presence of 1 µl/ml GolgiStop (BD Biosciences). The cells were then stained with anti-CD3 (BioLegend) and anti-CD8 (BD Biosciences) monoclonal antibodies for 30 min. Cells were fixed with Fixation and Permeabilization Solution (BD Biosciences) for 30 min at 4°C and permeabilized with Perm/Wash Buffer (BD Biosciences) for 20 min at room temperature. Cells were then stained for 30 min with anti-IL-17A antibody (BioLegend). Samples were collected with FACSCanto II and analyzed with FlowJo Software.

### Detection of T-Cell Subsets

For Treg cells, PBMCs were first stained with anti-CD3 antibody (BioLegend), then fixed and permeabilized. Cells were then stained with anti-FOXP3 antibody (BD Biosciences) for 1 hour before washing and acquisition. The T lymphocyte subpopulations were detected with the antibodies against CD3 (BioLegend), CD4 (BioLegend), CD8 (BioLegend), CD45RA (BD Biosciences) and CD27 (BD Biosciences). Cells acquisition was performed using a FACSCanto II flow cytometer, and the data were analyzed using FlowJo software.

### JAK or STAT3 Inhibitors Treatment *In Vitro*

HEK293 cells were transfected with the WT or mutant *STAT3* plasmid or empty vector. Forty-eight hours after the transfection, the cells were incubated for 4 hours in the presence of ruxolitinib (Abmole Bioscience), tofacitinib (Abmole Bioscience), stattic (Abmole Bioscience), or dimethyl sulfoxide (Sigma-Aldrich) before being activated with IL-6 (20 ng/mL) or not.

### Literature Review

The literature search was conducted in the PubMed, Web of Science, and Cochrane Library databases for relevant articles published until January 11, 2022, to identify studies reporting *STAT3* GOF germline mutations-related ILDs. All the search terms were Medical Subject Heading terms, including *STAT3* terms (“*STAT3*”, “signal transducer and activator of transcription 3”) and gain of function terms (“gain of function”, “activating germline mutation” and “activating mutation”). Additional studies were manually screened from citations of relevant articles. All selected articles were reviewed by abstract or full-text. Review articles, duplicate articles, and meeting abstracts were excluded.

### Statistics

Results were analyzed with the Student *t*-test using GraphPad Prism software, and *p* < 0.05 was considered statistically significant.

## Results

### Clinical Features of the Patient

**Figure 1:**
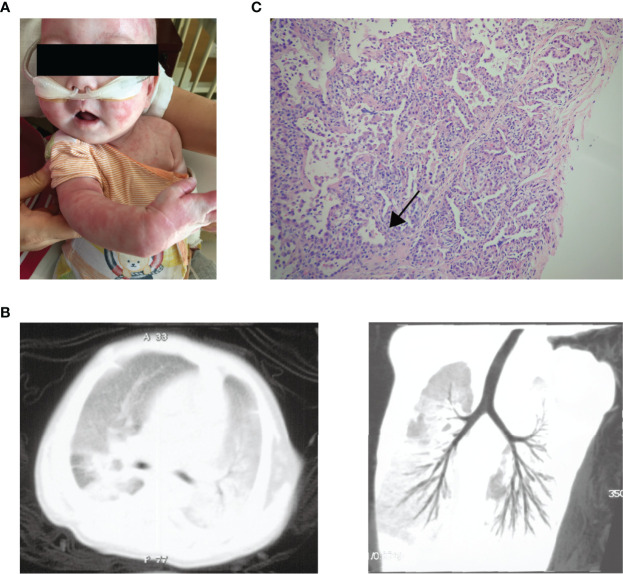
The clinical manifestations of the patient. **(A)** Generalised eczematous skin rashes in the patient. **(B)** Chest computed tomographic scan of the patient shows interstitial lung disease with diffuse ground-glass opacity. **(C)** Histology of lung biopsy from the patient. The alveolar membranes were markedly thickened (arrow) with macrophages, lymphocytes, and granulocytes in the alveolar cavity.

The patient was a 1-year-old boy of non-consanguineous Chinese parents. He was born at full-term with uneventful perinatal history. There was no family history of lung disease or primary immunodeficiency. He had no adverse reactions to vaccination with bacille Calmette-Guérin and hepatitis B. At 4 months of age, he was brought to the hospital for eczematous dermatitis (**Figure 1A**), mild cough, and a lump behind the left ear. His respiratory symptoms gradually worsened, and he developed respiratory distress and hypoxemia with possible cytomegalovirus (CMV) infection as the triggering event. Bronchoalveolar lavage and blood tests for CMV DNA were positive. He also suffered from repeated respiratory infections with positive sputum cultures of *Acinetobacter baumanni*, *Klebsiella pneumonia*, *Pseudomonas aeringinosa* and *Stentophomonas maltophilia*, and even twice respiratory failures requiring intubation and mechanical ventilation. Multiple courses of antibiotics have been required since then. He responded to the antibiotics with decreased inflammatory markers over 2-3 weeks, but fever recurred while on broad-spectrum antibiotics. At 6 months old, chest computed tomography suggested interstitial lung disease with diffuse ground-glass opacity (**Figure 1B**). The histology of lung biopsy showed chronic interstitial lung disease with diffuse thickening of alveolar membranes and hyperplastic type 2 pneumocytes (**Figure 1C**). The alveolar membranes contained some stromal cells and mild to moderate infiltrate of lymphocytes, some plasma cells, and macrophages. There was no proteinosis nor significant established interstitial fibrosis. Microbiological investigations of Lung biopsy including bacterial culture, fungal culture, acid-fast bacillus staining and PCR, and CMV were negative. Bronchoalveolar lavage pneumocystis pneumonia staining, blood tests for CMV, Epstein-Barr virus, human immunodeficiency virus, β-glucan and QuantiFERON-TB were negative. He was treated empirically with prednisolone (1.8/mg/kg/day) with gradual tapering according to the disease status. Hydroxychloroquine (6 mg/kg/day) was also added. The respiratory symptoms were partially improved. When chest symptoms were relatively stable, he also exhibited recurrent diarrhea with elevated white blood cells and neutrophils (**Table 1**) and one episode of stool culture positive for *Clostridium difficile*. At the age of 11months, he experienced increasing diarrhea, poor feeding, and septic shock with metabolic acidosis and elevated inflammatory markers. Unfortunately, he eventually died of respiratory failure at 1 year old.

**Table 1:** Laboratory parameters of the patient.

Laboratory parameters	Patient	Normal Range
**White Blood Cell (**x10^9^/L**)**	24.95	4-10
**Lymphocyte (**x10^9^/L**)**	7.62	3.73-8.76
**Neutrophil (**x10^9^/L**)**	15.06	1.4-6.5
**Hemoglobin (**g/L**)**	110	110-160
**Platelet Count (**x10^9^/L**)**	569	100-400
**Eosinophils (**x10^9^/L**)**	0.39	0.05-0.5
**Immunoglobulins**		
** IgG (**g/L**)**	7.27	5.0-10.6
** IgA (**g/L**)**	1.83	0.36-1.72
** IgM (**g/L**)**	1.36	0.44-2.07
** IgE (**IU/mL**)**	5	0.0-200.0
**Lymphocyte subset**		
** CD3^+^T cells (%)**	66.6	55.32-73.11
** CD4/CD8**	2.57	0.93-2.52
** CD19^+^B cells (%)**	30.7	17.20-29.71
** NK cells (%)**	0.6	5.67-15.90

### Identification of a Novel Heterozygous Mutation in the *STAT3* Gene

**Figure 2:**
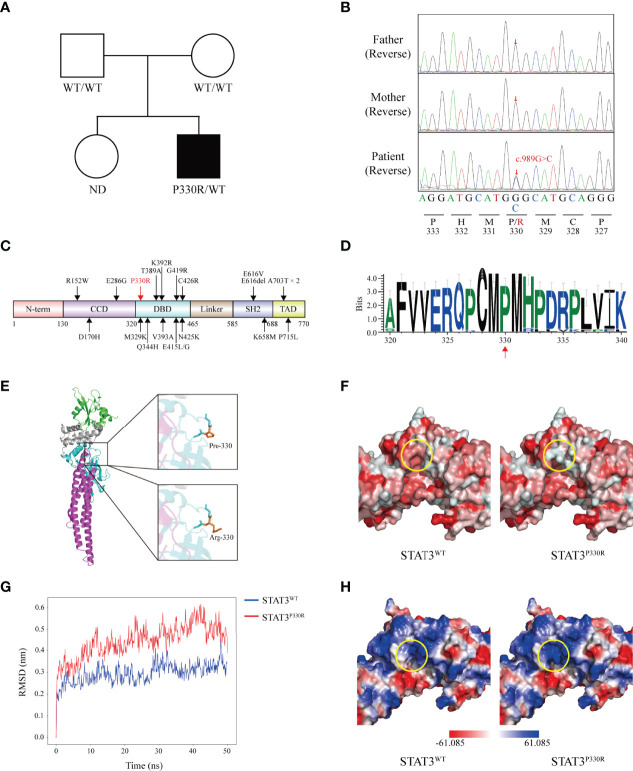
A novel *de novo* heterozygous mutation is identified in *STAT3.* **(A)** Pedigree of the affected family. Circles, females; squares, males; filled symbol, affected subject; open symbols, unaffected family members. ND, not determined. **(B)** Sequence analysis of the patient and his parents. The heterozygous single base substitution (c.989C>G, p.P330R) in *STAT3* is shown. **(C)** The schematic diagram shows the location of ILD-related GOF mutations on the STAT3 protein reported before. The position of the novel mutation identified in this study is marked in red. N-terminal domain (N-term), coiled-coil domain (CCD), DNA-binding domain (DBD), Linker domain (Linker), SH2-domain (SH2), and transactivation domain (TAD). **(D)** WebLogo demonstrates conservation of STAT3 amino acid sequence in 35 vertebrate species. **(E)** Crystal structure of the STAT3 (left) is shown in cartoon representation and colored as follows: CCD (magenta), DBD (cyan), Linker (grey), and SH2 (green). Magnified pictures displayed the sticks of Met329, Pro330 (top-right), Arg330 (bottom-right), and Met331 residue. Pro330 and Arg330 are colored in orange. **(F)** The predicted hydrophobicity (circled, yellow) of STAT3^WT^ and STAT3^P330R^. Gradient colors from red to white indicate a hydrophobicity scale from high to low. **(G)** The root-mean-square deviations (RMSDs) of the STAT3^WT^ and STAT3^P330R^ during 50 ns of molecular dynamics simulations. **(H)** The calculated electrostatic potentials of the STAT3^WT^ and STAT3^P330R^ are mapped on the surfaces and colored in a gradient from red (negative) to blue (positive).

Based on the clinical phenotype, congenital lung disease was considered for the patient. Genetic tests were done for the known related genes including *SFTPC*, *SFTPB*, *ABCA3*, *CSF2RA*, *CSF2RB*, *NKX2.1*, *FOXF1* and *SLC7A7*, but no meaningful mutation was revealed. Therefore, the next-generation sequencing was performed for the patient and his parents. No severe combined immunodeficiency-related genetic mutation was found. Instead, a *de novo* heterozygous missense mutation of the *STAT3* gene (c.989C>G, p.P330R) was identified in the patient (**Figures 2A, B**). The mutation was not found in the gnomAD (v2.1.1), ClinVar or Human Gene Mutation Database. This mutation was located within the DNA-binding domain (DBD), resulting in a proline to arginine substitution at position 330 (**Figure 2C**). The arginine residue was not observed at this position in homologous sequences (**Figure 2D**), suggesting highly conserved among different species.

Structurally, the proline is very rigid and therefore may induce a required exceptional backbone conformation at this position. To evaluate the impacts of this mutation on STAT3 structure, in silico studies were performed. The mutant residue lay on the surface of the STAT3 protein (**Figure 2E**) and decreased the hydrophobic interactions in this region (**Figure 2F**). We next performed the MD simulation of the WT and P330R STAT3 to estimate the conformational changes. The root-mean-square deviation (RMSD) for the P330R system showed higher fluctuations than the WT system (**Figure 2G**), suggesting an unstable protein structure. Beyond that, according to the electrostatic potential calculation, the charge changed from neutral (P) to positive (R) (**Figure 2H**), which may increase the interaction with negatively charged nucleic acids. These results strongly indicated that the mutation might alter the STAT3 structure and function.

### The Enhanced Phosphorylation and Transcriptional Activity of Mutant P330R STAT3

**Figure 3:**
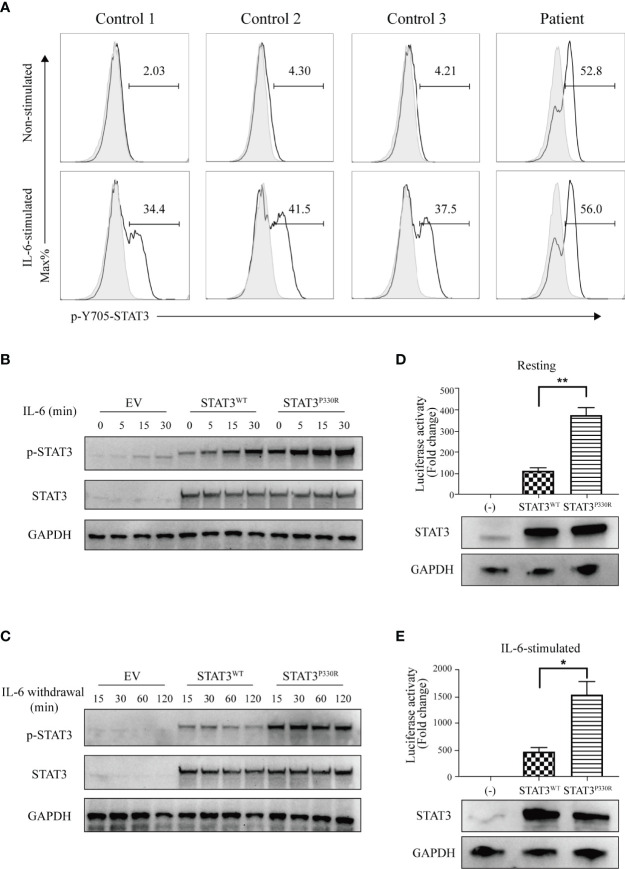
The mutation leads to increased phosphorylation and transcriptional activity. **(A)** Flow cytometric analysis of phosphorylated STAT3 in PBMCs from the patient and healthy controls with or without IL-6 stimulation. **(B, C)**. Western blot shows the phosphorylation **(B)** and dephosphorylation **(C)** of STAT3 in the human embryonic kidney 293 (HEK293) cells transfected with *STAT3* constructs. The total STAT3 and GAPPDH were used as the loading controls. EV, empty vector. The results are representative of three independent experiments, respectively. **(D, E)** The STAT3 relative luciferase activity in the absence **(D)** or presence **(E)** of IL-6 stimulation. The representative result from three independent experiments is shown, respectively. Relative STAT3 protein expression level was confirmed by western blotting. Data are mean  ± SEM (n = 3). *p < 0.05. **p < 0.01.

The functional alterations of mutant STAT3 were investigated. STAT3 can be activated *via* phosphorylation upon different cytokine stimulations, such as IL-6. It was demonstrated that, upon IL-6 stimulation, the patient cells have much higher phosphorylation of STAT3 compared to normal controls. In addition, the mutation also activated STAT3 under the steady-state (**Figure 3A**). Next, we further performed functional validation by transfecting either WT or mutant constructs into HEK293 cells. Like the findings in patient cells, the phosphorylated STAT3 of mutant *STAT3*-transfected cells was significantly enhanced than that of the WT after IL-6 stimulation. The STAT3^P330R^ mutant also displayed high phosphorylation in the basal condition (**Figure 3B**), suggesting constitutive activation. Besides, the P330R mutation also impaired the dephosphorylation of phosphorylated STAT3 on IL-6 deprivation (**Figure 3C**).

To detect the transcriptional activity of the P330R mutation, a STAT3-driven luciferase reporter assay was performed. We observed enhanced transcriptional activity of STAT3^P330R^ under the steady-state (**Figure 3D**), further suggesting constitutive activation of mutant STAT3. The mutant also showed a severalfold increase in the luciferase activity compared with STAT3^WT^ after being stimulated with IL-6 (**Figure 3E**). Collectively, these results suggested that the STAT3^P330R^ represented a GOF mutation leading to both increased phosphorylation and transcriptional activity of STAT3.

### The Increased Th17 Cells and Lymphocyte Subpopulation in the Patient

**Figure 4:**
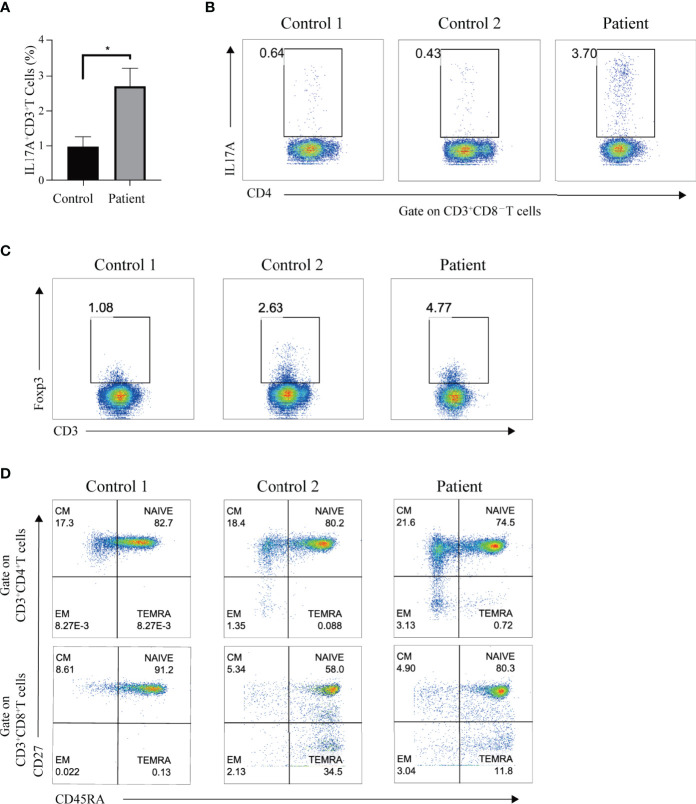
The Th17 cells and immune-phenotype in the patient. **(A)** The black and grey bars indicate the percentage of Th17 cells, as determined by flow cytometry, in phorbol myristate acetate (PMA)/ionomycin-activated PBMCs of the patient and healthy controls, respectively. Data are expressed as mean ± SEM. *p < 0.05. **(B)** The IL-17A production in T cells of the patient and two healthy controls after PMA/ionomycin treatment. **(C)** The representative dot plot shows the Treg population in the patient and two healthy controls, respectively. **(D)** Flow cytometry plots show the naive, central memory (CM), effector memory (EM), and terminally differentiated effector memory (TEMRA) CD4 or CD8 subpopulations in the patient and two age-matched healthy controls.

*STAT3* plays an irreplaceable role in regulating the immune signaling pathways, including inhibiting Tregs and enhancing Th17 cell determination (4). Given this, we investigated whether the P330R mutation would cause Th17 and Treg cells dysregulation. PMA/ionomycin-activated PBMCs of the patient showed increased IL-17A levels in the T cells compared to the normal controls (**Figures 4A, B**), suggesting the over-activation of the Th17 pathway. However, the percentage of Treg cells was within a normal range in the patient (**Figure 4C**). The levels of immunoglobulin G, A and M were not reduced. Lymphocyte subpopulation analysis indicated decreased NK cell percentage but normal T and B cell levels in the patient’s PBMCs (**Table 1**). The proportions of effector memory (EM) CD4^+^ T, EM CD8^+^ T, naive CD4^+^ T and naive CD8^+^ T cells were also normal (**Figure 4D**). Taken together, these data suggested that the STAT3^P330R^ gain-of-function mutation would contribute to activating the Th17 pathway.

### Both JAK Inhibitor and STAT3 Inhibitor Effectively Suppressed the Activation of STAT3^P330R^ Mutant

**Figure 5:**
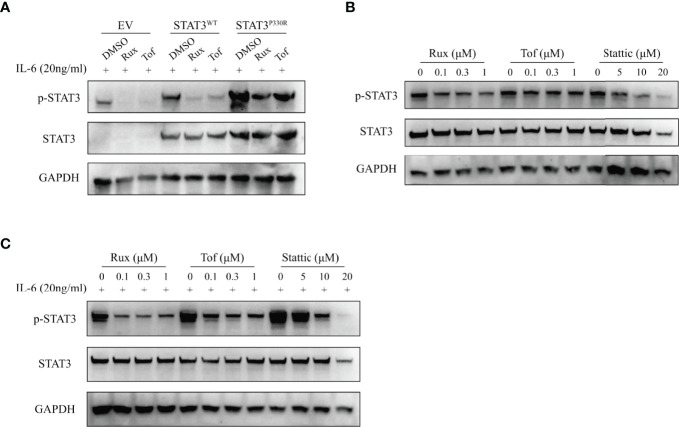
The suppression of STAT3 phosphorylation by JAK and STAT3 inhibitors. **(A)** The transfected HEK293 cells were treated with dimethylsulphoxide (DMSO), ruxolitinib (Rux), or tofacitinib (Tof), respectively, and then activated with IL-6 (20ng/ml). The phospho-STAT3 level was examined. **(B, C)** The transfected HEK293 cells were treated with ruxolitinib, tofacitinib or stattic at the indicated concentration and then activated without **(B)** or with **(C)** IL-6 (20ng/ml). The phosphorylation of STAT3 was examined by western blot. The total STAT3 and GAPPDH were used as the loading controls.

STAT3 is activated by JAK-mediated phosphorylation, so targeting upstream JAK kinases could be a potential approach to inhibit STAT3 activation. We thus performed functional experiments to determine whether JAK inhibitors could suppress STAT3^P330R^ activation. It was found that both ruxolitinib and tofacitinib significantly reduced the phosphorylation of mutant STAT3 induced by IL-6 stimulation (**Figure 5A**). Given the broad suppressive effect of JAK inhibitors, we sought to test the ability of a direct STAT3 selective inhibitor (Stattic) in pharmacologic inhibition of *STAT3* GOF mutation. Similar to JAK inhibitors, the STAT3 inhibitor also markedly suppressed the phosphorylation of mutant STAT3 in the steady-state, as well as in IL-6-stimulated condition (**Figures 5B, C**). In addition, the inhibitory effect was dose-dependent. The more amount of inhibitor used, the more inhibition of STAT3^P330R^ phosphorylation seen. These data indicated that selective inhibition of STAT3 represents a potential therapeutic approach for *STAT3* GOF syndrome.

### Literature Review of *STAT3* GOF Patients With ILD

Here we summarized current literature on the clinical features (**Table 2**) and treatments (**Table 3**) of *STAT3* GOF patients with ILD. Twenty patients were included, with 9 males, 10 females, and 1 gender not mentioned. In addition to ILD, all the patients also have other autoimmune disorders or lymphoproliferation. Eleven of 20 (55%) ILD patients had mutations in the DBD (**Figure 1C**). The median age at onset of ILD was 3 years based on the available information. One patient developed ILD at 3 months old, and prior to this, he was diagnosed with type I diabetes at 3 weeks of age. The most frequent lung disease was lymphocytic interstitial pneumonia (7/20, 35%), followed by desquamative interstitial pneumonia (3/20, 15%), nonspecific interstitial pneumonia (3/20, 15%) and interstitial pneumonia (3/20, 15%). The most common lung symptoms were dyspnea or tachypnea (7/20, 35%) and infections (6/20, 30%). Asthma was the primary manifestation before the diagnosis of ILD.

**Table 2:** Clinical characteristics of *STAT3* GOF patients with ILD.

Pt	Age of disease onset/Gender	Genotype	Pulmonary futures		Lymphoproliferative disease	Hematologic	Endocrine	Enteropathy	FTT	Inf.	Skin
			Type of ILD (Age at onset)	Others							
Pt 1 (This study)	4 m/M	c.989C>G; p.P330R	ILD (6 m)	Severe pneumonia	−	−	−	+	+	+	+
Pt 2 (3, 5)	At birth/F	c.1175A>G; p.K392R	DIP (<1 y)	Lower respiratory tract infections	ND	+	+	+	+	+	−
Pt 3 (4, 13, 14, 16)	3 y/F	c.1032G>C; p.Q344H	LIP (3 y)	Clubbing, tachypnea, on NIPPV and oxygen	+	+	−	+	+	+	−
Pt 4 (4)	< 1 y/F	c.2107G>A; p.A703T	LIP (ND)	ND	+	+	−	+	+	−	+
Pt 5 (4)	15 y/M	c.2107G>A; p.A703T	LIP (ND)	ND	+	+	−	−	ND	+	−
Pt 6 (13, 17)	3 y/M	c.1255G>C; p.G419R	Resembling DIP (13 y)	Dyspnea and cough	+	+	−	−	−	−	−
Pt 7 (9)	At birth/F	c.1847_1849delAAG; p.E616del;	LIP (< 2 y)	Severe respiratory infections	ND	ND	+	+	+	+	+
Pt 8 (14, 18)	1 y/F	ND; p.E415L	LIP (5 y)	Clubbing, tachypnea, retractions, hypoxia	+	ND	ND	+	+	ND	+
Pt 9 (16)	ND/M	c.2144C>T; p.P715L	LIP (ND)	LIP with restrictive disease and severe diffusion defect	+	+	+	+	+	−	+
Pt 10 (14, 19)	1 y/F	c.1178T>C; p.V393A	DIP (19 y)	History of asthma; clubbing, tachypnea, fine crackles	−	−	−	+	+	−	+
Pt 11 (7)	ND/F	c.454C>T; p.R152W	IP (ND)	ND	+	+	−	Not confirmed	+	+	ND
Pt 12 (12)	3 w/M	c.1973A>T; p.K658M	ILD (3 m)	Respiratory failure	ND	ND	+	+	+	−	ND
Pt 13 (14)	1 m/ND	ND; p.M329K	NSIP (7 y)	Clubbing, retractions, hypoxia, dyspnea with exertion	ND	ND	ND	ND	ND	ND	ND
Pt 14 (20)	7 m/M	c.508G>C; p.D170H	ILD (8 y)	Recurrent severe respiratory tract infections	+	−	+	+	+	+	ND
Pt 15 (21)	ND/M	ND; p.E286G	IP (ND)	Atypical pneumonia	−	+	−	−	−	ND	−
Pt 16 (21)	Infancy/F	ND; p.E415G	IP (> 2 y)	Recurrent respiratory infection	ND	+	+	+	+	+	+
Pt17 (13)	7 y/M	c.1165A>G; p.T389A	ILD (7 y)	History of asthma, respiratory failure	ND	ND	ND	ND	ND	ND	ND
Pt18 (15)	2 y/F	c.1847A>T; p.E616V	NSIP (2 y)	Dry cough, wheezing, hypoxia	+	−	−	−	−	−	+
Pt19 (15)	2 y/M	c.1276T>C;p.C426R	LIP (2 y)	Clubbing, tachypnea, retractions, hypoxia	+	−	+	+	+	+	+
Pt20 (15)	3 y/F	c.1275T>A; p.N425K	NSIP (3 y)	Cough, clubbing,tachypnea, retractions, hypoxia	+	−	−	+	+	+	+

DIP, desquamative interstitial pneumonia; F, Female; FTT, Failure to thrive; ILD, interstitial lung disease; IP, interstitial pneumonia; Inf., Infections; LIP, lymphocytic interstitial pneumonia; M, Male; ND, no data; NSIP, nonspeciﬁc interstitial pneumonia; NIPPV, noninvasive positive pressure ventilation; Pt, patient.

+, the presence of the symptom; − the absence of the symptom.

**Table 3:** Target therapies in *STAT3* GOF patients with ILD.

Patient	Agent	Dose	Improvement in lung symptoms	Complications	Follow-up period	Outcome
Pt 8 (14, 18)	Ruxolitinib	5 mg BID	Clubbing resolved, tachypnea and hypoxia improved	ND	2 y 1 m	Alive
Pt 10 (14, 19)	Tofacitinib	5 mg/d	Stable clubbing and tachypnea, crackles resolved	ND	1 y 6 m	Alive
Pt 13 (14)	Tofacitinib	10 mg BID	Stable clubbing and retractions, hypoxia and dyspnea resolved	ND	1 y 6 m	Alive
Pt 3 (4, 13, 14, 16)	First with tocilizumab	12mg/kg IV monthly	24hr BIPAP discontinued but still requires high flow NC during the day	ND	ND	Alive
	Then ruxolitinib	15mg BID	Clubbing improved, tachypnea resolved, off NIPPV and oxygen	Mild elevation in bilirubin	3 y 6 m	
Pt 9 (16)	First with ruxolitinib	7.5mg BID, then increased to 10mg BID	None	Raised ALP, ALT, and GGT	ND	Dead of respiratory failure complicated by pulmonary hemorrhage with aspergillosis, torulopsis glabrata, and pseudomonas sepsis.
	Then tocilizumab	400mg weekly increased to 500mg weekly	None	ND	ND	
Pt 11 (7)	First with tocilizumab	12 mg/kgr/15days	Yes	No	ND	Alive
	Then ruxolitinib	Initially 5 mg	Yes	No	ND	
Pt 12 (12)	Ruxolitinib	2.5 mg TID, 1.4 -2.2mg/kg/day	Yes	ND	10 m	Alive
	Tocilizumab	ND	Yes	ND	ND	

ALP, alkaline phosphatase; ALT, alanine transaminase; BID, twice a day; GGT, gamma-glutamyltransferase; NC, nasal cannula; ND, no data; Pt, patient; TID, three times a day.

As for the treatment, seven patients received targeted therapies: one received ruxolitinib only, two received tofacitinib only, and four received tocilizumab combined with ruxolitinib or tofacitinib. Only one patient died of respiratory failure, and a significant improvement was observed in the other six patients. Besides, the JAKs inhibitor was effective in patients who failed tocilizumab monotherapy.

## Discussion

In this study, we identified a novel *de novo* heterozygous *STAT3* mutation (c. 989C>G, p.P330R) in a patient with ILD, leading to increased phosphorylation and transcriptional activity of STAT3 and activated Th17 pathway. Unlike general *STAT3* GOF-related ILDs reported before, the case described here was infancy-onset with a fatal presentation. Apart from JAK inhibitors, the STAT3 selective inhibitor also effectively decreased the STAT3 phosphorylation caused by the P330R mutation.

*STAT3* GOF syndrome shows diverse clinical phenotypes in terms of the involved organs, predominant clinical manifestation and disease severity, which make the timely and correct diagnosis very challenging. Genetically, about half of the patients with ILD have mutations in the *STAT3* DNA-binding domain. However, no exact genotype-phenotype correlation has been defined so far. Clinically, *STAT3* GOF mutations cause multiple clinical manifestations appearing sequentially (6). The median onset age of ILD was the latest one amongst all the involved organs and showed slow progress in the majority of patients (6). But herein, we reported a patient with *STAT3* GOF mutation. He presented with infancy-onset ILD, and no other remarkable autoimmune disorders appeared before the ILD. In addition, the disease was severe and deteriorated in a short time, finally to death. Likewise, another patient with *STAT3* GOF mutation was recently described to have life-threatening infancy-onset ILD (12). However, different from our case, that patient also had another autoimmune disorder, type I diabetes, which developed prior to the ILD. Taken together, our study expanded the clinical spectrum of *STAT3* GOF syndrome, which could be an etiology of early-onset ILD. When early-onset ILD accompanied with or without other autoimmunities was encountered in the clinical setting, *STAT3* GOF mutation should be considered apart from other congenital pulmonary diseases. On the other hand, it seems that the progress of early-onset ILD is much faster and more severe in lung condition than other late-onset ILD. Therefore, the genetic test and functional validation should be performed immediately for the definitive diagnosis so that targeted therapy for the patient can be given timely to achieve a better outcome.

Unlike most previously reported *STAT3* GOF mutations (4, 5), the mutation in the *STAT3* DNA binding domain described here led to both increased transcriptional activity and phosphorylation of STAT3 in response to IL-6. In addition, this mutation also activated STAT3 in the resting condition, suggesting constitutive activation. Besides, in this study, delayed dephosphorylation kinetics was also observed after removing the IL-6 stimulation. Mertens et al. (22) demonstrate that mutations in the *STAT3* DNA-binding domain are crucial to controlling the DNA retention time. Structurally, the STAT3^P330R^ mutation changes a neutrally charged proline into a positively charged arginine, resulting in a stronger binding affinity to negatively charged DNA. These findings provide a possible explanation for the enhanced activity of mutant STAT3.

STAT proteins act as essential signal transducers in developing Th17 and Treg cells (23). *STAT3* promotes Th17 cell activation and expansion (24). In our patient with *STAT3* GOF mutation, the activation of mutant STAT3 was revealed in the steady-state. The patient also showed much higher phosphorylation of STAT3 than normal controls upon IL-6 stimulation. In parallel with this, Th17 cells were significantly higher in the patient than that in the controls, which may contribute to the autoimmune pathology of the patient. On the other side, STAT5 is a major transcriptional factor for Treg differentiation (24). A possible mechanism is proposed that STAT3 inhibits STAT5 phosphorylation *via* the suppressor of cytokine signaling 3, a negative regulator of STAT3 signaling, thereby inhibiting FOXP3 (4). Some patients with *STAT3* GOF mutation did show decreased STAT5 phosphorylation and Treg cells (4, 5, 21). However, the immune phenotypes of *STAT3* GOF mutations were not consistent in the previously described patients. Not all GOF mutations were found to affect STAT3 phosphorylation (4, 5). The patients had either increased, decreased, or normal Th17 cells. And Treg cells were reported to be decreased or not (4, 5, 8, 9). Thus, these data suggest that the mechanisms underlying the dysregulated Th17 and Treg cells in *STAT3* GOF syndrome are not clearly clarified and require further investigations.

So far, there is no standard treatment guideline available for *STAT3* GOF syndrome (10, 11). Nontargeted immunosuppressive therapies were found to be overall ineffective in *STAT3* GOF-related ILD, potentially increasing the risk of infections (12–15). The monotherapy with tocilizumab is also insufficient to improve respiratory status (14). JAK inhibitors were used successfully to treat *STAT3* GOF-related ILD and were also effective in patients who have failed tocilizumab monotherapy, whereas they did not reverse the pathologic scarring (14). Combining an anti-IL-6 receptor antibody and a JAK inhibitor was effective in ILD (7, 12, 14, 16). However, the pan-JAK inhibitors lacking pathway-selectivity raises a concern about the undesirable side effects. In our study, unfortunately, the patient died prematurely before the targeted therapy. *In vitro* experiments showed that the JAK inhibitors, both ruxolitinib and tofacitinib, significantly reduced IL-6-stimulated STAT3 phosphorylation. In addition, the STAT3 selective inhibitor, stattic, also suppressed the activation of mutant STAT3 in a dose-dependent pattern. Given the specificity of the STAT3 inhibitor, it would be one of the best candidate drugs for the targeted therapy and worth further *in vivo* studies to verify its potential.

In summary, in this study, we reported a novel *STAT3* GOF mutation in a patient with fatal infancy-onset ILD, which expanded the clinical spectrum of *STAT3* GOF syndrome. *STAT3* GOF mutation appears as a new etiology of ILDs and should be considered in patients with early-onset ILDs, especially those without known congenital pulmonary diseases. It needs to be acknowledged there could be additional genetic modifications that prone the patient for early demise. And the lack of evaluation of T cell function like proliferation test is a limitation of this study. In addition to JAK inhibitors, we also propose that the specific STAT3 inhibitor would be an appealing alternative for the precisely targeted therapies.

## Data AvAilability Statement

According to national legislation/guidelines, specifically the Administrative Regulations of the People’s Republic of China on Human Genetic Resources (http://www.gov.cn/zhengce/content/2019-06/10/content_5398829.htm, http://english.www.gov.cn/policies/latest_releases/2019/06/10/content_281476708945462.htm), no additional raw data is available at this time. Data of this project can be accessed after an approval application to the China National Genebank (CNGB, https://db.cngb.org/cnsa/). Please refer to https://db.cngb.org/, or email: CNGBdb@cngb.org for detailed application guidance. The accession code CNP0002801 should be included in the application.

## Ethics Statement

The studies involving human participants were reviewed and approved by the Institutional Review Board of the University of Hong Kong-Shenzhen Hospital. Written informed consent to participate in this study was provided by the participants’ legal guardian/next of kin. Written informed consent was obtained from the individual(s), and minor(s)’ legal guardian/next of kin, for the publication of any potentially identifiable images or data included in this article.

## Author Contributions

MD and YL (2nd author) performed the experiments and analyzed the data. MD wrote the manuscript. YL (3rd author) and XM screened the relevant literature. HK performed the molecular dynamics simulations guided by XL. WL collected and analyzed the clinical data guided by Y-LL. HM designed and guided the study and revised the manuscript critically. All authors contributed to the article and approved the submitted version.

## Funding

This study was supported in part by the National Key Research and Development Program of China (2021YFC2702005, National Natural Science Foundation of China (81971547; 21625201, 21961142010, 91853202; 81900136), the Research Fund for Outstanding Youth Scholar of Chongqing Talents (CQYC201905003), and the High-level Medical Reserved Personnel Training Project of Chongqing (2019181) and the Beijing Outstanding Young Scientist Program (BJJWZYJH01201910001001).

## Conflict of Interest

The authors declare that the research was conducted in the absence of any commercial or financial relationships that could be construed as a potential conflict of interest.

## Publisher’s Note

All claims expressed in this article are solely those of the authors and do not necessarily represent those of their affiliated organizations, or those of the publisher, the editors and the reviewers. Any product that may be evaluated in this article, or claim that may be made by its manufacturer, is not guaranteed or endorsed by the publisher.
